# An A_2A _adenosine receptor agonist, ATL313, reduces inflammation and improves survival in murine sepsis models

**DOI:** 10.1186/1471-2334-8-141

**Published:** 2008-10-20

**Authors:** Christopher C Moore, Edward N Martin, Grace H Lee, Tom Obrig, Joel Linden, W Michael Scheld

**Affiliations:** 1 Division of Infectious Diseases and International Health, Department of Medicine, University of Virginia, Box 801342, Charlottesville, VA 22908, USA; 2 Division of Nephrology, Department of Medicine, University of Virginia, Box 800133, Charlottesville, VA 22908, USA; 3 Cardiovascular Research Center, Department of Medicine and Pharmacology, University of Virginia, Box 801394, Charlottesville, VA 22908, USA

## Abstract

**Background:**

The pathophysiology of sepsis is due in part to early systemic inflammation. Here we describe molecular and cellular responses, as well as survival, in A_2A _adenosine receptor (AR) agonist treated and untreated animals during experimental sepsis.

**Methods:**

Sepsis was induced in mice by intraperitoneal inoculation of live bacteria (*Escherichia coli *or *Staphylococcus aureus*) or lipopolysaccharide (LPS). Mice inoculated with live bacteria were treated with an A_2A _AR agonist (ATL313) or phosphate buffered saline (PBS), with or without the addition of a dose of ceftriaxone. LPS inoculated mice were treated with ATL313 or PBS. Serum cytokines and chemokines were measured sequentially at 1, 2, 4, 8, and 24 hours after LPS was administered. In survival studies, mice were followed until death or for 7 days.

**Results:**

There was a significant survival benefit in mice infected with live *E. coli *(100% vs. 20%, *p *= 0.013) or *S. aureus *(60% vs. 20%, *p *= 0.02) when treated with ATL313 in conjunction with an antibiotic versus antibiotic alone. ATL313 also improved survival from endotoxic shock when compared to PBS treatment (90% vs. 40%, *p *= 0.005). The serum concentrations of TNF-α, MIP-1α, MCP-1, IFN-γ, and IL-17 were decreased by ATL313 after LPS injection (*p *< 0.05). Additionally, ATL313 increased the concentration of IL-10 under the same conditions (*p *< 0.05). Circulating white blood cell concentrations were higher in ATL313 treated animals (*p *< 0.01).

**Conclusion:**

Further studies are warranted to determine the clinical utility of ATL313 as a novel treatment for sepsis.

## Background

Approximately 900,000 cases of sepsis occur annually in the United States, causing roughly 210,000 deaths and costing almost 17 billion dollars [1]. The overwhelming inflammation that occurs along with infection during sepsis has been the target of several therapeutic interventions [2]. Unfortunately, despite successful treatment in animal models, antibody neutralization of individual components of this inflammation has not proved beneficial for the majority of patients in clinical sepsis trials [3].

Tissue hypoxia, as occurs in sepsis, enhances breakdown of adenosine triphosphate (ATP) to adenosine monophosphate (AMP), which is then dephosphorylated by the cytosolic 5'nucloeotidase to adenosine [4]. Adenosine can bind to four G protein coupled receptors, A_1_, A_2A_, A_2B_, and A_3_. The A_2A _adenosine receptor (AR) is present on inflammatory cells including neutrophils, mast cells, monocytes, macrophages, eosinophils, platelets, and T-cells, and is involved in anti-inflammatory activities [5]. Activation of A_2A _ARs results in an increase in cyclic AMP concentration in inflammatory cells which is increased further by concurrent type IV phosphodiesterase inhibitors. A_2A_AR agonists decrease superoxide production in neutrophils, degranulation of neutrophils, TNF-α production by monocytes and macrophages, and neutrophil-endothelial cell adherence [6]. Death occurs in mice deficient for *Adora2a*, the A_2A _AR gene, after exposure to Concanavalin A liver injury within 8 hours compared to complete survival in wild-type mice. Pro-inflammatory cytokines are present in higher concentrations in *Adora2a*^-/- ^mice when compared to wild-type mice. Similar findings are observed in experimental animals exposed to LPS [7].

To assess the broad applicability of A_2A _AR agonists, specifically ATL313, in the treatment of experimental sepsis due to different infections we contrasted survival results in mice challenged with a gram-negative (*Escherichia coli*) vs. gram-positive (*Staphylococcus aureus*) pathogen or purified LPS. T cells are increasingly recognized as important in the pathogenesis of experimental and clinical sepsis [8]. Cytokine expression, inflammation, and therefore outcomes may differ during experimental sepsis depending upon the mouse strain and its T cell repertoire. This may have implications for sepsis therapy including ATL313. Therefore, we used a mouse strain, C57BL/6, with a predominantly Th1 phenotype and a mouse strain, Balb/C, with a predominantly Th2 phenotype to see if there were differences in outcomes based on underlying T cell phenotypes [9].

Sepsis starts as a process of intravascular inflammation mediated by pro-inflammatory cytokines/chemokines including TNF-α, MIP-1α, MCP-1, IFN-γ, and IL-17 as well as anti-inflammatory cytokines, e.g. IL-10 [10]. Therefore, to better understand the underlying protective effect of A_2A _AR agonists, we evaluated cytokines in animals undergoing experimental sepsis with and without the addition of ATL313. ATL313 is a hundred fold more selective for the A_2A _AR than for the A_1 _AR and twenty fold more selective than for the A_3 _AR. Furthermore, ATL313 is more selective and has a longer half-life (approximately 30 minutes in rodents) than its A_2A _AR agonist predecessors. The A_2A _AR agonists are potentially useful therapeutic agents because, unlike nonspecific AR agonists, A_2A _AR agonists do not induce hypotension [11]. We also studied the peripheral blood of the animals to assess the impact of an A_2A _AR agonist on circulating white blood cell concentrations.

## Methods

### Mice

Female C57BL/6 and BALB/c mice (≅20 g; Jackson Laboratories, Bar Harbor, ME) were housed at 68–72°F with a 12 h light/dark cycle, fed standard laboratory food and water *ad libitum*, and were kept under specific pathogen-free conditions. The protocol used in this study was approved by the Animal Care and Use Committee of the University of Virginia.

### Reagents and drugs

LPS (*E. coli *O111:B4) was purchased from Sigma (St. Louis, MO). The A_2A _AR agonist, ATL313, (4-{3-(6-amino-9-(5-cyclopropylcarbamoyl-3,4-dihydroxytetrahydrofuran-2-yl)-9H-purin-2-yl)prop-2-ynyl}piperidine-1-carboxylic acid methyl ester) was supplied by Adenosine Therapeutics, LLC (Charlottesville, VA). Ketamine and xylazine were purchased from Vedco, Inc., (St. Joseph, MO). In all cases vehicle used was phosphate buffered saline (PBS).

### Dose effect

The appropriate dose of ATL313 for survival studies was determined by a dose response curve where increasing doses of ATL313 (5–200 μg/kg) were administered every 6 hours to female BALB/c and C57BL/6 mice after the intraperitoneal (IP) injection of a lethal dose of O111:B4 LPS (20 mg/kg). A similar dose response curve was used to determine the optimal dose of LPS for BALB/c and C57BL/6 mice.

### Survival studies

Female C57BL/6 mice were inoculated with 1 × 10^8 ^colony forming units (CFU) live K12, O26:B6 *E. coli *or 8 × 10^8 ^CFU *S. aureus *at t = 0 and inoculated with the A_2A _AR agonist, ATL313 (5 μg/kg), or PBS, at t = 8, and then every 6 hours spanning 48 hours. To determine an optimal dosing interval, ATL313 was administered every 12 hours spanning 48 hours for *S. aureus *inoculated mice. A dose of ceftriaxone (25 mg/kg; t = 8) was given to a subset of treated and control mice to create a total of four experimental groups (bacterial infection + PBS; bacterial infection + ATL313; bacterial infection + Ceftriaxone; bacterial infection + ATL313 + Ceftriaxone). Female BALB/c mice were also tested in the *E. coli *model (data not shown). Female BALB/c mice undergoing endotoxemia were injected IP with LPS one half hour prior to injection with ATL313 (5 μg/kg) or PBS which occurred at t = 0 and every 6 hrs thereafter, for a total of eight doses spanning 48 hours (N = 20–29 per group). To provide equal fluid resuscitation in the setting of sepsis, all injections were I mL in volume. Moribund mice were anesthetized using ketamine and xylazine and euthanized via cervical dislocation.

### Time course studies

Female C57BL/6 and BALB/c mice were injected intraperitoneally (IP) with O111:B4 LPS and ATL313 or PBS as described above. Experimental and control animals were sequentially sacrificed at times 1, 2, 4, 8, and 24 hours after LPS injection. The mice were anesthetized with ketamine and xylazine and killed by cervical dislocation. Immediately prior to sacrifice the animal underwent cardiac puncture and ventricular blood was aspirated for white blood cell analysis via the Hemavet 850 veterinary multi-species hematology system (Drew Scientific, Inc., Oxford, CT) and future cytokine analysis.

### Cytokine quantification

Cytokine investigations were carried out using a protein bead-based multiplex immunoassay system (Bio-Rad Laboratories, Hercules, CA). With this system we are able to measure IL-1α and β, TNF-α, IFN-γ, IL-2, IL-3, IL-4, IL-5, IL-6, IL-10, IL-12 p40 and p70, IL-17, KC, MIP-1α, and RANTES through use of pre-packaged bead arrays. This panel allows for evaluation of early and late appearing as well as pro- and anti-inflammatory cytokines.

### Statistical analysis

Statistical comparisons of cytokine values were done by a 2 tailed Student's t-Test (Microsoft Excel software, Microsoft Corporation, Redmond, WA). Survival data were plotted and the survival curves compared with a log-rank test (GraphPad PRISM software, San Diego, CA). Data are displayed as means ± SEM unless otherwise stated. Differences were considered significant at p < 0.05.

## Results

### ATL313 improves survival in three sepsis models

**Figure 1:**
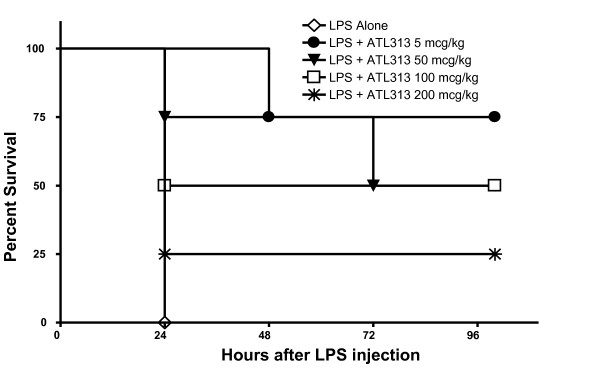
**Dose effect.** The optimal dose of ATL313 was determined through a dose response curve where PBS vehicle (white diamond), 5 μg/kg ATL313 (black circle), 50 μg/kg ATL313 (black triangle), 100 μg/kg ATL313 (white square), or 200 μg/kg ATL313 (asterix) was injected IP every 6 hours into BALB/c mice (N = 4 per group) after a lethal IP dose of LPS O111:B4 (20 mg/kg).

**Figure 2:**
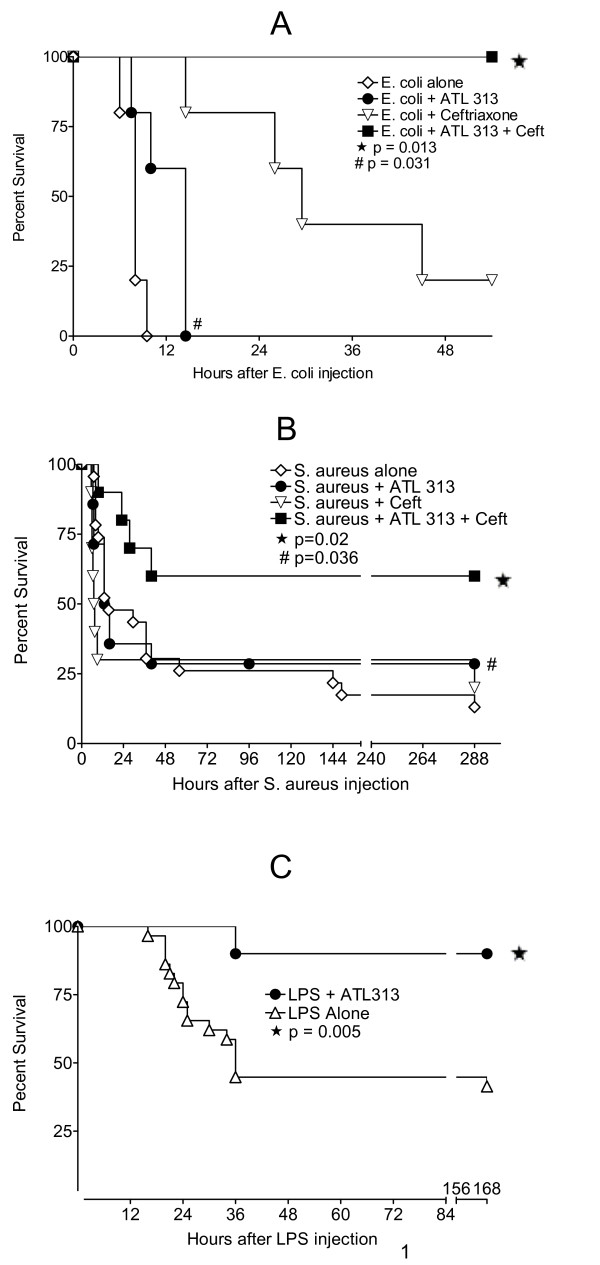
**ATL313 protects mice in three different sepsis models.** ATL313 protects mice from live *E. coli *challenge. Mice were injected with 1 × 10^8 ^CFU of live *E. coli*. Mice (N = 5 per group) were treated with PBS vehicle (white diamond), ATL313 (black circle), the antibiotic ceftriaxone (white triangle), or a combination of ceftriaxone and ATL313 (black square). Ceftriaxone was administered at a single dose of 25 mg/kg eight hours following inoculation. ATL313 (5 μg/kg) or PBS was dosed IP starting 8 hours after *E. coli *injection then every 6 hours spanning 48 hours. ATL313 + ceftriaxone treatment protects mice better than ceftriaxone treatment alone (* p = 0.013) and treatment with ATL313 alone prolongs life compared to *E. coli *untreated controls (#p = 0.031) (A). ATL313 protects mice from live *S. aureus *challenge. Mice were injected IP with 8 × 10^8 ^cfu of live *S. aureus*. Mice (N = 10–24 per group) were treated with PBS vehicle (white diamond), ATL313 (black circle), the antibiotic ceftriaxone (white triangle), or a combination of ceftriaxone and ATL313 (black square). Ceftriaxone was administered at a single dose of 25 mg/kg eight hours following inoculation. ATL313 (5 μg/kg) or vehicle was dosed IP starting at eight hours after *S. aureus *injection 4 times at 12 hour intervals. ATL313 + ceftriaxone treatment protects mice better than ceftriaxone treatment alone (* p = 0.02) and ATL313 treatment alone increases survival in *S. aureus *untreated controls (#p = 0.036) (B). ATL313 decreases LPS-induced mouse mortality via A_2A _AR-mediated mechanisms. Mice (N = 20–29 per group) were injected IP with LPS from *E. coli *(O111:B4, 5 mg/kg). One half hour prior to LPS challenge and at 6 hr intervals, PBS vehicle (white triangle) or ATL313 (black circle) was injected (5 μg/kg) IP for a total of 8 doses/48 hr. ATL313 protects the mice compared to LPS-challenged mice in the absence of ATL313 (* p = 0.005) (C).

The optimal dose of ATL313 for survival studies was 5 μg/kg in BALB/c (Figure 1) and C57BL/6 (data not shown) mice. The survival rate for the four groups inoculated with live *E. coli *were: PBS alone, 0%; ATL313 alone, 0% (p = 0.031 when compared to *E. coli *alone as time to death was prolonged); Ceftriaxone alone, 20%; Ceftriaxone plus ATL313, 100% (*p *= 0.013 when compared to ceftriaxone treatment alone) (Figure 2A). A second group of mice were injected IP with live *S. aureus*. The survival of mice treated with the combination of ceftriaxone and ATL313 was 60%, compared to 20% of mice receiving ceftriaxone alone (*p *= 0.02) and 13% of vehicle controls (*S. aureus *vs. *S. aureus *+ ATL313, *p *= 0.036) (Figure 2B). Finally, the survival rate over 7 days of mice injected IP with LPS was approximately 40%. ATL313 dosed at six hour intervals beginning one half-hour before LPS increased LPS challenged mouse survival to approximately 90% (*p *= 0.005) (Figure 2C).

### ATL313 influences circulating cytokine concentrations after LPS challenge

**Figure 3:**
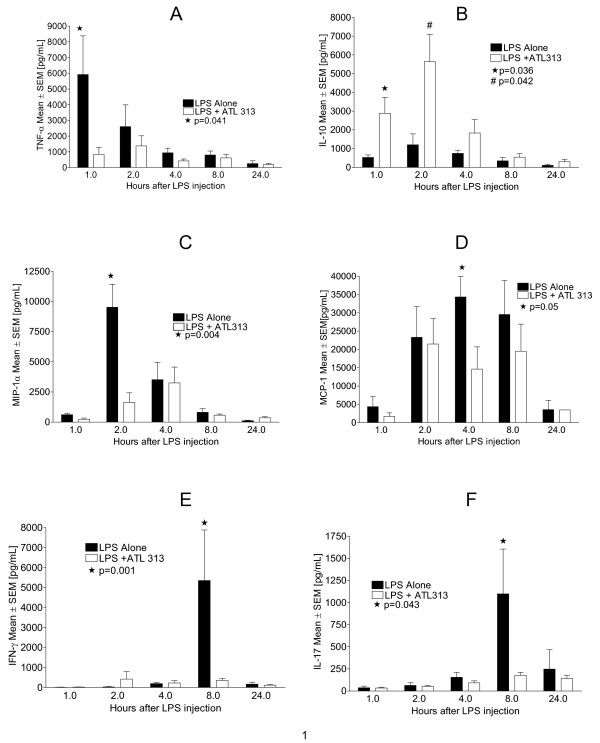
**ATL313 affects circulating cytokines and chemokines. **The A_2A _AR agonist, ATL313 significantly decreases the concentrations of pro-inflammatory cytokines and chemokines in mice (N = 5–9 per group) after inoculation with *E. coli *O111:B4 LPS (25 mg/kg). Mean ± standard error of the mean concentrations are shown in pg/mL. In ATL313 treated animals, TNF-α, at t = 1 hour, was significantly lower than in untreated animals (* p = 0.041) (A). Conversely, the anti-inflammatory cytokine, IL-10, had an increase in concentration in ATL 313 treated animals at t = 1 and 2 hours (* p = 0.036; # p = 0.042) (B). Like TNF-α, MIP-1α concentrations are increased early on after LPS exposure and significantly decreased by ATL313 at t = 2 hours (* p = 0.004) (C). Increases in MCP-1 concentrations occur later after exposure to LPS and are significantly decreased by ATL313 at t = 4 hours (* p = 0.05) (D). Both IFN-γ and IL-17 concentrations are maximal at t = 8 hours and are significantly attenuated by ATL313 at that time (* p = 0.001 and 0.043 respectively) (E and F).

Individual cytokine concentrations displayed statistically significant differences at different intervals after treatment with ATL313 when compared to controls. In plasma, at time (t) = 1 hour, TNF-α concentrations were significantly higher in untreated animals (N = 7) than treated animals (p = 0.041) (Figure 3A). Conversely, IL-10 in plasma was significantly lower at 1 and 2 hours after LPS inoculation in untreated animals vs. treated animals (p = 0.036 and 0.042 respectively) (Figure 3B). MIP-1α is also attenuated early on by ATL313. At t = 2 hours, the untreated animals had a higher concentration of MIP-1α in plasma than those treated with ATL313 (p = 0.004) (Figure 3C). MCP-1 values were significantly lower for ATL313 treated animals at t = 4 hours (p = 0.05) (Figure 3D). IFN-γ appeared later and was significantly higher at t = 8 hours in animals exposed only to LPS than in treated animals (p = 0.001) (Figure 3E). IL-17 was also decreased by ATL313. The difference was statistically significant at t = 8 (p = 0.043) (Figure 3F). Although differences between untreated and treated mice were observed, these did not reach statistical significance for the following cytokines/chemokines: IL-1α, IL-1β, IL-2, IL-3, IL-4, IL-5, IL-6, IL-12 p40, IL-12 p70, KC, and RANTES (data not shown). These analytes were chosen to allow assessment of early and late, and pro- and anti-inflammatory cytokines.

### ATL313 increases peripheral white blood cell concentrations in endotoxemic mice

**Figure 4:**
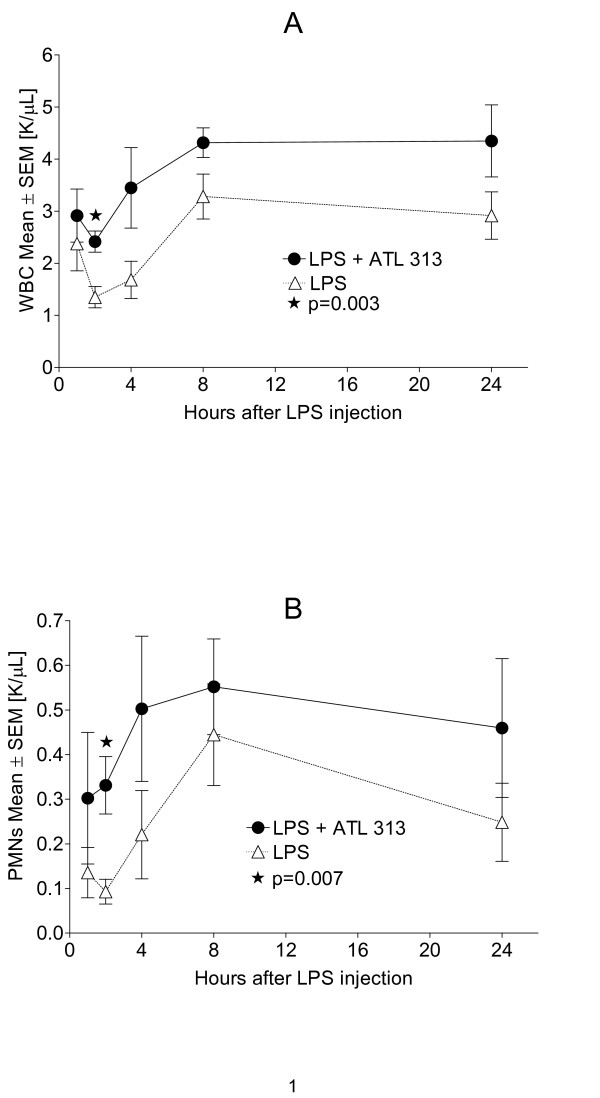
**ATL313 affects circulating white blood cells.** ATL313 (black circle) increases concentrations of circulating white blood cells in mice (N = 6 per group) after inoculation with LPS from *E. coli *(O111:B4, 5 mg/kg) (A). Additionally, circulating neutrophil (PMN) concentrations are also increased in mice treated with ATL313 (black circle) after LPS inoculation (B). Mean ± standard error of the mean concentrations are shown in K/μL. The difference in concentrations is significantly different at t = 2 hours for both total WBC and PMN concentrations (* WBC p = 0.003; PMN p = 0.007).

During experimental endotoxemia in BALB/c mice we found that the concentration of circulating white blood cells (WBCs) was higher in ATL313 treated animals when compared to untreated animals. This difference was consistent at all time points and statistically significant at t = 2 hours after LPS injection (p = 0.003) (Figure 4A). This finding was mirrored by the population of neutrophils (PMNs) in the blood. The concentration of PMNs in the blood was also consistently higher at all time points and statistically significantly higher after 2 hours in animals treated with ATL313 (p = 0.007) (Figure 4B). As in the case of total WBCs and PMNs, the trend towards higher concentration of peripheral blood cells across all time periods in treated animals was true for lymphocytes as well. At t = 8, the concentration difference between untreated and treated (N = 6) resulted in a p value = 0.05 (data not shown).

## Discussion

This study documents: 1) a survival benefit with administration of ATL313 during live E. coli and S. aureus sepsis (in conjunction with an antibiotic) and in LPS-induced sepsis; 2) a decrease of TNF-α, MIP-1α, MCP-1, IFN-γ, and IL-17 blood concentrations in animals receiving A_2A _AR agonists with a concordant increase in IL-10 concentrations; and 3) a relative increase in circulating peripheral blood leukocyte concentrations in mice receiving ATL313 when compared to controls. The first observation bears on the generalizability of our observations to different etiologies of sepsis, including both gram positive and gram negative infections, while the latter two emphasize the mechanism(s) of protection. While these results are encouraging, further work is needed to prove the efficacy of these compounds in clinical sepsis and other models of sepsis including cecal ligation and puncture.

In our murine model of LPS-induced sepsis, TNF-α blood concentrations rise rapidly and are profoundly decreased by the co-administration of an A_2A _AR agonist, ATL313. We have previously shown that the administration of an A_2A _AR agonist up to 24 hours after LPS challenge is still protective (i.e. improved survival) when TNF-α concentrations in blood have returned to baseline; therefore the benefit cannot be attributed to effects on TNF-α alone [12]. Given the same argument, it is unlikely that the decrease in MIP-1α, MCP-1, IFN-γ or IL-17 completely explains the protective efficacy of A_2A _AR agonists in experimental murine sepsis. However, the effect of A_2A _AR agonists on multiple cytokines is encouraging, since the clinical presentation of sepsis in humans is highly variable with regard to the cytokine cascade, but other as yet undetermined targets may play a role as well.

Inoculation of experimental animals with TNF-α alone reproduces many of the fundamental pathophysiologic alterations typical of sepsis [13]. In vivo, TNF-α alters endothelium and is a potent chemoattractant for neutrophils which contributes to the pathology of venous thromboses, arteriosclerosis, vasculitis, and disseminated intravascular coagulation [14,15]. By decreasing TNF-α expression, A_2A _AR agonists may decrease neutrophil recruitment and therefore inflammatory damage to the endothelium. This conjecture is bolstered by the increased number of neutrophils in the circulation of mice treated with ATL313 after LPS challenge. Our findings are mirrored by in vitro and in vivo work which revealed that A_2A _AR agonists decreased neutrophil extravasation and their release of oxidative and non-oxidative products in experimental gram negative bacterial meningitis and *S. aureus *septic arthritis [16,17].

Cytokines can cause inflammation via upregulation of other pro-inflammatory cytokines in the case of TNF-α, MIP-1α and IL-17, modulation of T-cells by IFN-γ, and activation of monocytes by MCP-1 [18-21]. These pro-inflammatory mechanisms result in recruitment and activation of neutrophils, NK cells, and macrophages which produce deleterious reactive oxygen species and lysosomal enzymes [22-25]. TNF-α, MIP-1α, MCP-1, and IFN-γ have been measured in patients with sepsis and acute respiratory distress syndrome and are correlated with poor outcomes [14,26,27]. Antibodies to MIP-1α, IL-17, and IFN-γ have ameliorative in vivo effects in animal sepsis studies [28-32]. As in the case of TNF-α, A_2A _AR agonists down-regulate the expression of MIP-1α, MCP-1, IFN-γ, and IL-17 with a concurrent survival benefit.

While pro-inflammatory cytokines such as TNF-α, MIP-1α, MCP-1, IFN-γ, and IL-17 increase the inflammatory response, anti-inflammatory cytokines such as IL-10 temper this response. IL-10 is produced primarily by Th-2 cells and decreases concentrations of TNF-α by degrading cytokine mRNA [33]. IFN-γ production is thought to be inhibited by IL-10 in a more indirect manner through the suppression of IL-12 production [34].

IL-10 reduces the release of TNF-α into the circulation which hinders the development of a systemic inflammatory response syndrome, and this correlates with an increase in survival [35-38]. We have found that elevated IL-10 and decreased TNF in the first few hours of experimental sepsis is correlated with increased survival. It is possible that the decrease in pro-inflammatory cytokines occurs as a direct result of the increase in IL-10 when an animal is treated with ATL313. Given the changes in IL-10 expression in endotoxemic mice after treatment with an A_2A _AR agonist in C57BL/6 mice, we studied survival in a predominantly Th2 T-lymphocyte populated mouse, e.g. BALB/c. Again, we showed a significant survival benefit in these animals treated with an A_2A _AR agonist after live *E. coli *or LPS O111:B4 injection.

Therefore, the benefit of ATL313 in these sepsis models is equally beneficial in mice with a Th1 or Th2 phenotype, perhaps implying that ATL313 and similar compounds exert their anti-inflammatory effect through modification of the innate immune system. We are actively pursuing this line of investigation. Additionally, targeting individual cytokines alone has not been clinically successful, but a more pluripotent approach such as is offered by A_2A _AR agonists may be more successful. The influence of A_2A _AR agonists on late mediators including triggering receptor expressed on myeloid cells (TREM-1) and High mobility group box 1 (HMGB1) should be investigated and is the subject of our ongoing research efforts.

## Conclusion

Collectively, we have shown that the effect of A_2A _AR agonists spans different mouse strains, different LPS preparations, as well as different formulations of A_2A _AR agonists. We also investigated a model of *S. aureus *sepsis and saw a survival benefit after treatment with ATL313 and ceftriaxone when compared to ceftriaxone alone. A_2A _AR agonists provide an avenue to study the inflammation due to sepsis and should be considered for clinical interventions in septic patients. Ultimately, it will be important to delineate which cytokines and cells are the effectors of this anti-inflammatory effect.

## Competing interests

WMS and JL have equity interests in Adenosine Therapeutics, LLC who provided the A_2A _Adenosine Receptor agonist compound, ATL313

## Authors' contributions

CCM designed and performed all experiments and drafted the manuscript. ENM assisted in design and performance of some experiments. GL performed some experiments. TO assisted in design of some experiments. JL assisted in design of some experiments. WMS oversaw all elements of experimental design and execution and manuscript preparation.

## Pre-publication history

The pre-publication history for this paper can be accessed here:


